# How the Impact of Median Neuropathy on Sensorimotor Control Capability of Hands for Diabetes: An Achievable Assessment from Functional Perspectives

**DOI:** 10.1371/journal.pone.0094452

**Published:** 2014-04-10

**Authors:** Haw-Yen Chiu, Hsiu-Yun Hsu, Li-Chieh Kuo, Fong-Chin Su, Hui-I Yu, Shih-Che Hua, Chieh-Hsiang Lu

**Affiliations:** 1 Section of Plastic Surgery, Department of Surgery, Chi-Mei Medical Center, Tainan, Taiwan; 2 Department of Physical Medicine and Rehabilitation, National Cheng Kung University Hospital, College of Medicine, National Cheng Kung University, Tainan, Taiwan; 3 Department of Biomedical Engineering, National Cheng Kung University, Tainan, Taiwan; 4 Department of Occupational Therapy, National Cheng Kung University, Tainan, Taiwan; 5 Medical Device Innovation Center, National Cheng Kung University, Tainan, Taiwan; 6 Department of Internal Medicine, Ditmanson Medical Foundation, Chiayi Christian Hospital, Chiayi City, Taiwan; 7 Department of Business Administration, College of Management, National Chung Cheng University, Chiayi County, Taiwan; 8 Ta Tung Institute of Commerce and Technology, Chiayi City, Taiwan; Banner Alzheimer's Institute, United States of America

## Abstract

To comprehend the sensorimotor control ability in diabetic hands, this study investigated the sensation, motor function and precision pinch performances derived from a pinch-holding-up activity (PHUA) test of the hands of diabetic patients and healthy subjects. The precision, sensitivity and specificity of the PHUA test in the measurements of diabetic patients were also analyzed. We hypothesized that the diabetic hands would have impacts on the sensorimotor functions of the hand performances under functionally quantitative measurements. One hundred and fifty-nine patients with clinically defined diabetes mellitus (DM) and 95 age- and gender-matched healthy controls were included. Semmes-Weinstein monofilament (SWM), static and moving two-point discrimination (S2PD and M2PD), maximal pinch strength and precision pinch performance tests were conducted to evaluate the sensation, motor and sensorimotor status of the recruited hands. The results showed that there were significant differences (all *p*<0.05) in SWM, S2PD, M2PD and maximum pinch strength between the DM and control groups. A higher force ratio in the DM patients than in the controls (*p*<0.001) revealed a poor ability of pinch force adjustment in the DM patients. The percentage of maximal pinch strength was also significantly different (*p*<0.001) between the DM and control groups. The sensitivity, specificity and area under the receiver operating characteristic curve were 0.85, 0.51, and 0.724, respectively, for the PHUA test. Statistically significant degradations in sensory and motor functions and sensorimotor control ability were observed in the hands of the diabetic patients. The PHUA test could be feasibly used as a clinical tool to determine the sensorimotor function of the hands of diabetic patients from a functional perspective.

## Introduction

Diabetes mellitus (DM) is a chronic illness that affects multiple organs and systems. Various functional and pathological changes occur in type 2 diabetes patients as a consequence of glycemic derangement [1], [2]. Diabetic neuropathy (DN), a related micro-vascular complication [3], has been reported to have negative impacts on the performance of daily living activities [4]. While DN includes a host of diabetic neuropathic syndromes, chronic sensorimotor polyneuropathy, characterized by the presence of symptoms of peripheral nerve dysfunction, is the main clinical form [5]. The clinical features of chronic sensorimotor polyneuropathy are progressive and often start in the feet. The reported occurrence rate of DN ranges from 28.5% to 90% [6], [7], [8]. While numerous investigations on diabetes have analyzed the corresponding symptoms with regards to pain, tingling, paresthesia and sensory loss in the lower extremities [9], few have examined neuropathies in the hands, as these are reported less often by diabetic patients.

A restricted range of motion, insufficient muscle strength, and carpal tunnel syndrome have been reported to be the main manifestations of DN in the hand [10], [11]. Recently, sensorimotor disturbances in diabetic hands were reported to be an important factor in compromising the hand functions of such patients [12]. Redmond et al. reported that the tactile sensation in diabetic hands was abnormal with reduced function over a two-year follow-up period [13]. Some studies have also reported abnormal neurophysiological findings in the median nerves of diabetic patients [14], [15]. In addition, a recent study reported that 16.8% of asymptomatic diabetic hands met the inclusion criteria for subclinical median neuropathy, including abnormal nerve conduction velocity and an enlarged cross-sectional area of the carpal tunnel [16]. In addition, a previous nerve conduction study reported that the prevalence of subclinical neuropathy was 58% to 82% in the median nerves of diabetic patients [17]. Since asymptomatic neuropathy progresses as the number of nerve fibers gradually reduces, a sensitive and precise evaluation tool to detect neuropathy in the early stage is critical in a clinical setting. While sensory and motor nerve conduction studies are recommended as the gold standard to determine peripheral neuropathy in the hand [18], [19], these are costly and time consuming [20]. While quantitative sensory tests such as the Semmes-Weinstein monofilament (SWM) test can be used to identify sensory neuropathy earlier, they only have moderate sensitivity and specificity [12], [21]. Further, there are currently no appropriate tools to explore sensorimotor control in diabetic hands.

The ability of pinch force adjustment to changes in the inertial load has been analyzed in a precision pinch experimental model in order to better understand sensorimotor control in the hand [22], [23]. A recent study reported that sensory-deficient patients had problems in generating grip forces efficiently when handling an object [24]. Partial impairment of tactile sensibility, which affects motor efficiency when executing functional tasks, has been observed in several previous studies [25], [26]. A recent study reported that the force ratio derived from a custom pinch-holding-up activity (PHUA) test is a reliable tool to determine the characteristics of sensorimotor control in the hand [27]. Since the ability to use the hand well involves mobility, strength, sensation and coordination [28], the precision pinch model can help clinicians to assess hand manipulation objectively among patients with impaired sensibility [29].

Since chronic sensorimotor polyneuropathy has the strongest negative impact on the health-related quality of life of diabetic patients [4], early detection and appropriate management are very important. Although many quantitative sensory tests have been used to detect neuropathy in diabetic hands [30], the sensorimotor control of the hands of patients with DN has yet to be investigated. Therefore, the primary aim of this study was to identify the sensation, motor function and sensorimotor control ability of the hands of diabetic patients without neurological symptoms. The secondary aim was to examine the sensitivity and specificity of the PHUA test in the diagnosis of diabetic sensorimotor neuropathy.

## Methods

### Subjects

The participants in this prospective case-control study included clinically defined diabetic patients who were diagnosed based on the 1997 criteria of the American Diabetes Association [31], and controls matched by age, gender, and handedness. None of the enrolled diabetic subjects reported experiencing any hand discomfort. The patients with (1) diagnosed neuro-musculoskeletal disorders, (2) traumatic nerve injuries of the upper limbs, (3) trauma to the hand or congenital anomalies of the wrist and hand, (4) skin infections or disease, or (5) cognitive deficits, were excluded from the sample. A total of 170 DM patients were initially screened and referred by the Division of Metabolism and Endocrinology outpatient clinic from a teaching hospital in southern Taiwan over a period of 6 months. Eleven of them, however, have experienced previous hand trauma and cervical radiculopathy so that were excluded from this study. There were finally 159 diabetic patients who fulfilled the inclusion criteria in our patient group. The recruited patients were all right handed. Ninety-five volunteer control subjects were recruited from the local community, matched according to age, sex, and handedness. None of the control subjects had any sensory disturbances in their hands or any related neurological disorders. The demographic and clinical characteristics of both the control and patient groups are listed in Table 1. The mean age of the patient group was 58.83±9.64 years, and 58.70±10.94 years for the control group, and there was no statistically significant difference between the groups.

**Table 1:** Demographic characteristics of the 159 diabetic patients and 95 control subjects.

	Control subjects (n = 95)	Diabetic patients (n = 159)
Gender (Male:Female)	48∶47	83∶76
Age (years)	58.70±10.94	58.83±9.64
	NGSP HbA1C (%)	7.66±1.33
	IFCC HbA1C (mmol/mol)	60.17±14.48
	AC sugar	138.1±47.1
	Total cholesterol (mmol/l)	173.2±34.0
Clinical characteristics of the diabetic patients (n = 159)	Triglycerides (mmol/l)	124.6±75.6
	High-density lipoprotein	54.0±14.1
	Low-density lipoprotein	104.4±30.3
	Creatinine	1.15±0.77
	Weight (kg)	69.0±12.4
	Height (cm)	161.4±8.3

### Ethics Statement

All participants were informed about the purpose of the study and signed consent forms. The study was approved by the Institutional Review Board at Ditmanson Medical Foundation, Chiayi Christian Hospital.

### Instruments

#### Pinch Apparatus

The pinch apparatus (dimension: 6×4.5×9 cm; weight: 480 g) used in this study has been described in detail in our previous report [27]. Two 6-axis load cells (force/torque transducers: Nano-25; ATI Industrial Automation, Apex, NC) and an accelerometer (Model 2412; Silicon Designs, Inc., Issaquah, WA) were embedded in the apparatus to register the online pinch force exerted by the subjects and the acceleration of the pinch apparatus in space, respectively. According to Newton's second law, the measured load force equals the product of mass (m) and the vector summation of gravity (g) and the lifting acceleration (a). The pinch force and the load force were recorded and computed during the performance of discrete vertical movements in order to measure the capacity of adjusting pinch force according to inertial load. A high reliability has been reported for repeated measurements of the PHUA test (intra-correlation coefficient values of 0.84∼0.96) [27].

#### Sensibility Tests

Semmes-Weinstein monofilament (SWM), static two-point discrimination (S2PD) and moving 2PD (M2PD) tests were used to evaluate the sensory status of the hands of the recruited subjects. The S2PD and M2PD tests examine the innervation density of slowly and quickly adapting nerve fibers, respectively, and their corresponding mechanoreceptors. S2PD tests were used to detect the shortest distance between points that the subjects could perceive, and SWM tests were used to determine the touch-pressure threshold of the hands. When carrying out the SWM test, the filament exerts a constant force onto the skin area, and the monofilament is labeled with a numerical marking which is a log to the base ten of the force in tenths of a milligram.

### Procedures of the PHUA Test (Fig. 1A)

Before performing the PHUA test, the subjects first washed their hand with soap and water to remove any greasy substances. Verbal instructions were given to the subjects regarding the timing of task sequences prior to the experiment. The subjects were asked to pinch and lift the apparatus using the pulps of their thumb and index finger to about 5 cm above the table, with the forearm extending forward in an upright sitting posture. They held the apparatus at this position for 5 seconds, and then lifted the apparatus to a height of 30 cm, and then slowly lowered it to its initial position. The data collection period for each trial was 15 seconds. In addition, the subjects were instructed to pinch and lift the apparatus gently at a self-paced lifting speed. The participants were allowed three practice trials and the examiners checked their performance carefully to ensure that they completely understood the PHUA test before formal data recording. The test procedures were repeated three times for each hand, with a 1-minute resting interval between each trial. The data from three trials were averaged for analysis. After three trials of the test, the subjects performed a pinch with maximal pinch force exertion at a height of 30 cm above table to determine the static maximal pinch force.

### Data Processing and Analysis

**Figure 1:**
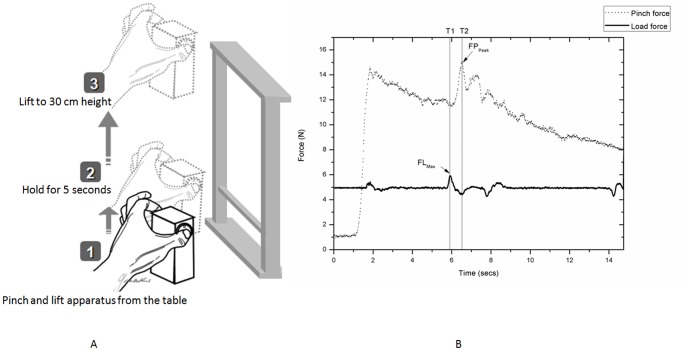
The experimental procedures and data analysis used in this study. (A): the procedures; and (B): data analysis of the PHUA test.

The ability of pinch force adjustment according to the inertial load is shown in Fig. 1B. Data analysis was performed at two distinct time points; T1 (maximum upward acceleration during the lifting-up phase), and T2 (peak pinch force during the lifting-up phase). The following parameters of the PHUA task were analyzed (Fig. 1B): (1) FP_Peak_: maximum pinch force during the lifting phase in the PHUA test; (2) FL_Max_: maximum load force at the onset of maximum upward acceleration; (3) force ratio of FP_Peak_ to FL_Max_; (4) percentage of maximal pinch strength: peak pinch force divided by the maximal static pinch force as a percentage of maximum voluntary contraction; and (5) time lag: the time latency between T1and T2 (Fig. 1B), which was the temporal coupling between FP_Peak_ and FL_Max_ during the PHUA test. A custom-made Matlab computer program was used to compute these parameters. The discriminative sensory function was assessed by determining the minimum distance the subjects could detect in the static and moving 2PD tests. The force in grams determined from the SWM test was defined as the pressure-threshold of the hands.

### Experimental Protocol

The basic personal information, clinical characteristics of the diabetic patients, results of the 2PD and SWM tests, and temporal and force parameters obtained from the PHUA test were recorded for all participants.

### Statistical Analysis

SPSS 17.0 for Windows (Statistical Package for Social Sciences Inc. Chicago, IL, USA) was used for all statistical analyses. Descriptive statistics were used to describe the clinical characteristics of the diabetic patients, results of the S2PD, M2PD, and SWM tests, static maximum pinch strength (N), and force parameters of the PHUA test. The independent *t*-test was used to test the differences in the results obtained from the diabetic and control groups. The level of significance was set at *p*<0.05. Receiver operating characteristic (ROC) curves were constructed using sensitivity and specificity values from the results of the force ratio derived from the PHUA test of the patients and healthy controls to determine the cutoff values. The cutoff scores for the subtests were determined by the Youden index. To determine the accuracy of the PHUA test, the area under the ROC curve (AUC) was calculated.

## Results

### Clinical Characteristics of the Participants

The clinical characteristics of the study population are summarized in Table 1. There were 83 males and 76 females with a mean age of 58.83 years. In addition, 48 males and 47 females with a mean age of 58.70 years were recruited into the control group. The mean length of time from the first diagnosis of diabetes was 114.6 months (range 4 months to 324 months). The glycosylated hemoglobin level of the 159 recruited patients ranged from 5.1% to 13.5% (32 to 124 mmol/mol), with a mean value of 7.66±1.33% (60.17±14.48 mmol/mol).

### Sensory and Motor Function of the Median Nerves

**Figure 2:**
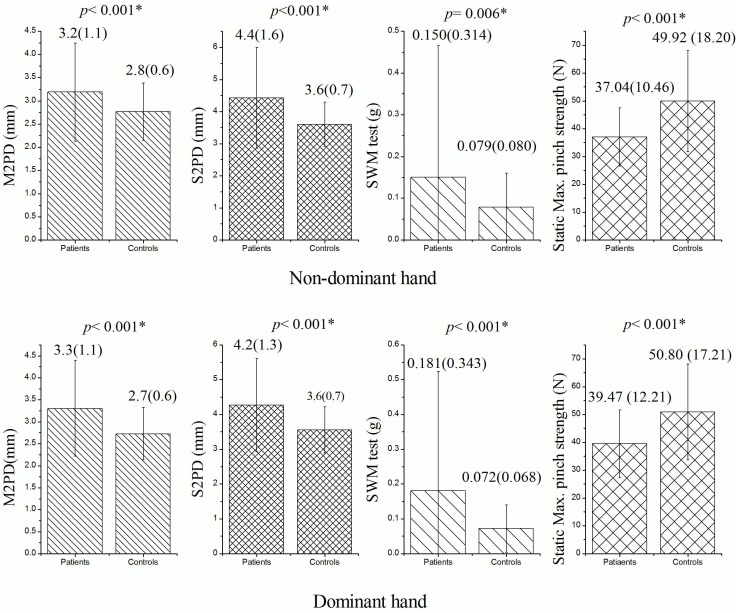
Mean (±SD) of M2PD, S2PD, SWM and static maximum pinch strength for the 159 diabetic patients and 95 control subjects. (Statistical analysis method: independent t-test; the level of significance was set at *p*<0.05.)

Figure 2 shows that there was a statistically significant difference (*p*<0.001) in the static and moving 2PD results of both hands between the diabetic and control groups. In addition, there were also statistically significant differences in the SWM results for the non-dominant (*p* = 0.006) and dominant hands (*p*<0.001) of the diabetic and control groups. With regards to the motor function of the median nerve, the static maximum pinch strength results for the patients and controls were 37.04± 10.46 N and 49.92 ±18.20 N, respectively, in the non-dominant hands, and 39.47±12.21 N and 50.80±17.21 N, respectively, in the dominant hands. The differences in static maximum pinch strength between the diabetic patients and control subjects were significant for both the dominant and non-dominant hands (both *p*<0.01).

### Precision Pinch Performance Detected by the PHUA test

**Figure 3:**
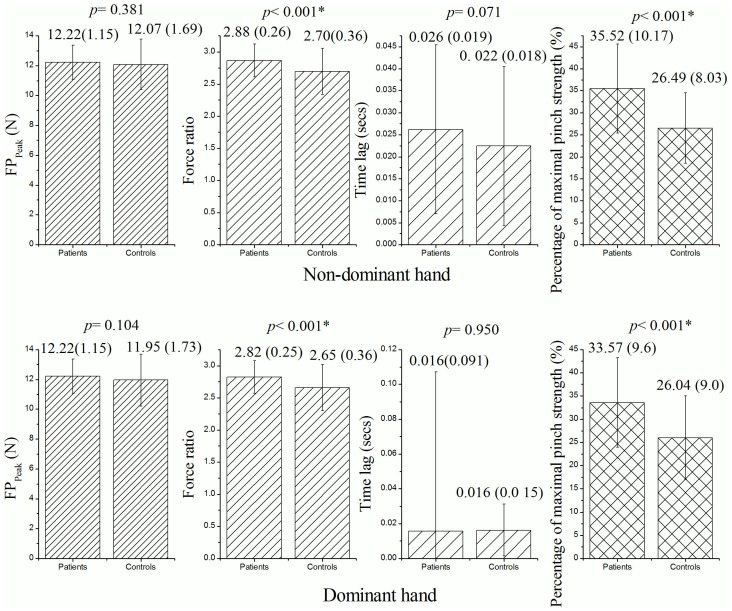
Mean (±SD) of outcome parameters derived from the PHUA test between the 159 diabetic patients and 95 control subjects. (Statistical analysis method: independent *t*-test; the level of significance was set at *p*<0.05.)

There were statistically significant differences in the force ratio (*p*<0.001) and percentage of maximal pinch strength (*p*<0.001) of both hands between the diabetic and control groups (Fig. 3). The force ratios for the diabetic and healthy subjects were 2.88±0.26 and 2.70±0.36, respectively, in the non-dominant hands, and 2.82±0.25 and 2.65±0.36, respectively, in the dominant hands. In addition, the percentage of maximum pinch strength was higher for both hands in the diabetic patients (35.52±10.17% and 33.57±9.6%, respectively for the non-dominant and dominant hands) compared to the healthy controls (26.49±8.03% and 26.04±9.0%, respectively, for the non-dominant and dominant hands). With regards to the FP_Peak_ values, the pinch force generated in both hands by the diabetic patients did not differ significantly from that for the healthy controls in the lifting task (*p* = 0.381 and *p* = 0.104, respectively, for the non-dominant and dominant hands). A similar trend was found for the time lag between pinch and load coupling (*p* = 0.071 and *p* = 0.950, respectively, for the non-dominant and dominant hands) (Fig. 3).

### ROC Curves of Functional Sensibility

**Figure 4:**
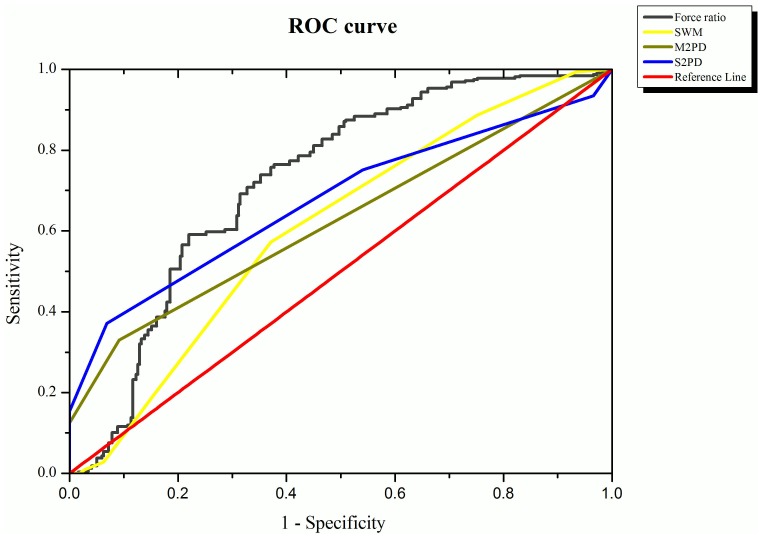
The ROC curves of the 2PD, SWM and PHUA tests for the diabetic patients.

The sensitivity, specificity, and optimal cutoff points calculated for the PHUA, S2PD, M2PD and SWM tests are shown in Figure 4 and Table 2. The sensitivity was 0.85 and specificity 0.51 for the force ratio in the PHUA test, with a suggested score of 2.61 for force ratio as the optimal cutoff point when screening for the diabetic patients. The cutoff points of the reference tools, SWM, S2PD and M2PD, were 0.048 g, 4.5 mm and 3.5 mm, respectively. According to the AUC of the force ratio (0.724), the PHUA test is thus a sensitive tool for diagnosing diabetic neuropathy. Compared to the traditional sensibility tests, the AUC of the force ratio was higher than the AUC of the SWM (0.519), M2PD (0.626) and S2PD (0.674).

**Table 2:** Area under the curve, sensitivity, specificity and cutoff points of the 2PD, SWM and PHUA tests for the diabetic patients.

	Area under the curve	Sensitivity	Specificity	Optimal cutoff point
M2PD (mm)	0.626	0.33	0.90	3.5
S2PD (mm)	0.674	0.55	0.43	4.5
SWM (g)	0.519	0.55	0.43	0.048
Force ratio	0.724	0.85	0.51	2.61

## Discussion

To clarify whether compromised sensation, motor function and sensorimotor control ability of hands are due to the complications of micro-vascular damage in diabetic patients, the threshold and discriminative sensibility, static maximum pinch strength and efficiency of adjusting pinch force in the PHUA test were analyzed for 159 diabetic patients and 95 healthy subjects in this study. A statistically significant difference in the touch-pressure threshold as measured by the SWM test between the patients and healthy controls was observed in both the dominant (*p* = 0.006) and non-dominant (*p*<0.001) hands. The degradation in the threshold sensation of the hands of the diabetic patients is consistent with a number of earlier studies [10], [32]. The results of the discriminative sensory function as measured by moving and static 2PD, confirmed that our patient group had worse sensation in the hands compared to the control group. To the best of our knowledge, no previous research has used the S2PD and M2PD tests to explore the discriminative sensation in the hands of diabetic patients. Nevertheless, the clinical findings with regards to sensory neuropathy in the diabetic hands in this study are not the same as sensory results for early carpal tunnel syndrome [33], as patients with the latter condition only experience deficits in threshold tests. One reason for this may be that most of the diabetic patients recruited in the current study suffered from chronic neuropathy, which leads to deficits in discriminative sensation related to demyelination or axonal injury of the median nerve.

With regards to the motor function results, there was a significant difference between the two groups with regards to static maximum pinch strength (*p*<0.001). More specifically, the pinch strength was significantly lower in the diabetic group compared with the control group, consistent with the findings of Cetinus et al [2]. The sensory function and muscle strength results of the current study revealed that the neuropathy of the diabetic patients affected both motor and sensory fibers in the hands. This supports the findings of a previous study [34], in which chronic denervation in the hands of diabetic patients was found based on nerve conduction and electromyography.

The efficiency of pinch force exertion analyzed by a dynamic pinch-lifting task is adjusted automatically based on pre-programmed muscle commands and online sensory information, but not the conscious control of the subject [35]. The ability to adjust pinch force in relation to the inertial load of the pinch apparatus can thus be considered to be an index of sensorimotor control of the hands. The force ratio of FP_Peak_ to FL_Max_, was much higher (*p*<0.001) in the diabetic patients than in the controls, supporting the hypothesis that the ability to adjust pinch force according to the load of the object being handled is adversely affected due to micro-vascular complications in diabetic patients. However, the difference in the FP_Peak_ (peak pinch force) for both hands between the diabetic patients and healthy controls did not reach a significant level. The reduced static pinch strength of the diabetic patients may be the main reason for this. The percentage of maximum voluntary contraction (peak pinch force divided by maximal static pinch force) was thus an important parameter that could be used to remove variations in pinch strength between the two groups in the current study. According to the results, the percentage of maximal pinch strength was statistically and significantly higher (*p*<0.001) in the diabetic group compared to the control group. This means that the diabetic patients exerted a higher percentage of maximum pinch force to maintain a stable pincer grip in the PHUA test, which is consistent with the findings of a previous study [36]. A recent study regarding the assessment and intervention of hands with sensorimotor deficits reported that the ability to adjust balanced pinch force in the PHUA test correlated significantly with hand function [29], and thus we can infer that poor hand function is more likely to develop in diabetic patients with peripheral neuropathy.

To examine the discriminative ability of the PHUA test in diagnosing diabetic neuropathy, ROC curves were constructed using the sensitivity and specificity values of force ratio derived from this test [37]. The AUC for the reference scales, SWM, S2PD and M2PD tests, were 0.519, 0.674 and 0.626, respectively. However, the AUC for the force ratio was much higher at 0.724. This shows that the PHUA test has a better ability to differentiate sensorimotor deficits in the hands of diabetic patients from healthy controls than the other tests. As for the better classification of diabetic and healthy hands, the force ratio derived from the PHUA test had a higher sensitivity (0.85) than the SWM (0.55) and 2PD tests (0.55 and 0.33, respectively, for S2PD and M2PD). A previous study demonstrated that the sensitivity of the 10-g SWM test was 53.8% in the feet of asymptomatic patients, with a specificity of 100% [38]. However, the sensitivity and specificity of the SWM test for differentiating diabetic hands in the current study were only 0.55 and 0.43, respectively. Although the SWM test is recommended to identify patients suffering from the risk of foot ulcers, it is not precise enough to detect neuropathy in the hands. Unlike traditional sensibility tests (e.g., the SWM and 2PD tests) which only detect the density of specific mechanoreceptors, the use of the dynamic pinch model is related to both the integrated impulses transmitted from the sensory receptors and motor execution. Therefore, it is likely to have better sensitivity and accuracy in determining asymptomatic neuropathy in diabetic hands.

According to a previous study that examined the hand function of diabetic patients, finger dexterity tests did not seem to be precise enough with regards to identifying differences in hand dexterity between groups with type 2 DM and normal glucose tolerance [12]. In this study, the diabetic patients mostly prevented objects slipping from their hands in a rather crude manner. With regards to sensorimotor control of the hands, similar results have been found in subjects with carpal tunnel syndrome [24] and in those with digital anesthesia [39]. However, no previous studies have investigated sensorimotor control in the hands of a diabetic population using a dynamic coordination model. Based on the results of this study, precision pinch performance can be analyzed in order to identify sensorimotor control of the hand, an issue that is of significant clinical relevance for diabetic patients. This is the first investigation to reveal specific deficits in the domains of sensation, motor function and sensorimotor control with regards to diabetic hands. In addition, the results of this study demonstrate the accuracy and feasibility of the PHUA test in determining asymptomatic neuropathy for diabetic patients. However, there are still several limitations in this study. One of them is the capability to carry out the dynamic manipulations among the patients with various severities of neuropathy based on the gold standard of a nerve conduction study has not been investigated in the current study. In addition, there was still lack of the investigation of the relationships between the results of PHUA and factors regarding hand dexterity for the DM patients. Future works thus require exploring the associations among hand control ability, actual median nerve status and hand functions in diabetic patients so that the clinicians could make proper treatment strategies accordingly.

## Conclusions

This study demonstrated that the diabetic patients had problems with regards to impaired sensation, motor function and sensorimotor control. It also validated the accuracy of the force ratio derived using a precision pinch model to examine sensorimotor control of the hand. Diabetes not only has the potential to develop into carpal tunnel syndrome, but also into subclinical and sensorimotor neuropathy, which has been underestimated in some reports. All diabetic patients should thus be screened with regards to neuropathy at an early stage in order to prevent further complications in the hands, such as trigger fingers, range of motion limitations and infection. The results of this study also show that the PHUA test is a sensitive and precise tool that can used in practice for the early detection of neuropathy in diabetic hands.
